# The Characterization of a Gonococcal HicAB Toxin–Antitoxin System Capable of Causing Bacteriostatic Growth Arrest

**DOI:** 10.3390/microorganisms13071619

**Published:** 2025-07-09

**Authors:** Salwa S. Bagabas, Jorge Trujillo-Mendoza, Michael J. Stocks, David P. J. Turner, Neil J. Oldfield

**Affiliations:** 1 Department of Biological Sciences, Faculty of Science, University of Jeddah, Jeddah 23445, Saudi Arabia; salwa.hz.sb@gmail.com; 2 School of Life Sciences, University of Nottingham, Nottingham NG7 2RD, UK; jorge.trujillomendoza@nottingham.ac.uk (J.T.-M.); david.turner@nottingham.ac.uk (D.P.J.T.); 3 School of Pharmacy, University of Nottingham, Nottingham NG7 2RD, UK; michael.stocks@nottingham.ac.uk

**Keywords:** *Neisseria gonorrhoeae*, toxin–antitoxin, HicA, HicB, HicAB, growth arrest, biofilm, bacteriophage

## Abstract

*Neisseria gonorrhoeae* is the causative agent of the sexually transmitted infection gonorrhea. Preventative vaccines or novel treatments based on a better understanding of the molecular basis of *N. gonorrhoeae* infection are required as resistance to current antibiotics is widespread. Toxin–antitoxin (TA) systems modulate bacterial physiology by interfering with vital cellular processes; type II TA systems, where both toxin and antitoxin are proteins, are the best-studied. Bioinformatics analysis revealed genes encoding an uncharacterized type II HicAB TA system in the *N. gonorrhoeae* strain FA1090 chromosome, which were also present in >83% of the other gonococcal genome sequences examined. Gonococcal HicA overproduction inhibited bacterial growth in *Escherichia coli,* an effect that could be counteracted by the co-expression of HicB. Kill/rescue assays showed that this effect was bacteriostatic rather than bactericidal. The site-directed mutagenesis of key histidine and glycine residues (Gly22, His24, His29) abolished HicA-mediated growth arrest. *N. gonorrhoeae* FA1090∆*hicAB* and complemented derivatives that expressed IPTG-inducible *hicA*, *hicB*, or *hicAB*, respectively, grew as wild type, except for IPTG-induced FA1090∆*hicAB::hicA.* RT-PCR demonstrated that *hicAB* are transcribed in vitro under the culture conditions used. The deletion of *hicAB* had no effect on biofilm formation. Our study describes the first characterization of a HicAB TA system in *N. gonorrhoeae*.

## 1. Introduction

The Gram-negative bacterium *Neisseria gonorrhoeae* (the gonococcus) causes gonorrhea, one of the most common sexually transmitted infections (STIs) worldwide [1]. Gonorrhea commonly presents as urethritis in men and cervicitis in women, but infections can be asymptomatic. If not detected or adequately treated, gonorrhea infection in women can lead to complications such as salpingitis, pelvic inflammatory disease, chronic pelvic pain, ectopic pregnancy, and infertility [2]. Additionally, gonococcal infection can be transmitted vertically during vaginal delivery causing neonatal conjunctivitis and is associated with an increased risk of mother-to-child HIV-1 transmission [3,4]. Treatment is by antibiotic therapy, but resistance to multiple classes of antibiotics has emerged, leaving few effective treatment options [5,6]. Currently gonorrhea prevention relies largely on promoting safe sexual behaviours and regular screening for STIs [2].

Toxin–antitoxin (TA) systems are small genetic modules that are widely distributed in the chromosomes of prokaryotes, as well as in mobile genetic elements (MGEs) such as plasmids and phages [7]. TA systems consist of a pair of genes encoding a toxin targeting an essential cellular process (such as transcription, translation, cell division, or membrane integrity) and an antitoxin that can limit toxicity. TA system toxin activities are tightly controlled at multiple levels but, under certain conditions, sufficient free active toxin becomes available for toxicity effects on growth to be discerned [8]. TA systems have been suggested to play roles in maintaining MGEs, stress responses, biofilm formation, and the mediation of phage resistance [9,10]. TA systems differ in terms of genetic organization, toxin targets, mechanism of toxin neutralization, and regulation; eight TA system types (types I-VIII) are currently recognized [11]. In type I-VII TA systems, the toxins are proteins, whereas, in type VIII systems, they are RNAs. In type I, III, and VIII TA systems, antitoxins are small non-coding RNAs while in type II, IV, V, VI, and VII TA systems, the antitoxins are small proteins [11]. Type II systems, where both the toxin and antitoxin are proteins which interact to form a non-toxic complex, are thought to be the most abundant and diverse [12]. Based on structural similarities, type II toxins have been classified into superfamilies including ParE/RelE, MazF, VapC, Zeta, and HicA [13]. Typically, each toxin superfamily is associated with a specific antitoxin superfamily, but hybrid associations have been described [14,15].

HicAB systems are predicted by genomic analysis to be abundantly present across free-living archaea and bacteria [16,17], but remain poorly studied [18]. Only a few HicAB systems, notably from *Escherichia coli* [17,19], *Burkholderia pseudomallei* [20,21], *Yersinia pestis* [22], and *Streptococcus pneumoniae* [23], have been experimentally characterized. In general, HicA toxins are small (50–100 amino acids) monodomain proteins, which harbour a double-stranded RNA-binding domain (dsRBD fold) and degrade RNAs. However, their substrate specificities and recognition motifs are unclear [16,18]. HicB antitoxins have a conserved partial RNase H fold in their *N*-terminus (which neutralizes HicA), and either an HTH or RHH DNA binding domain at their *C*-terminal end (allowing HicB and HicAB complexes to repress their own promoters) [22,23,24]. Unlike many other type II TA systems, the antitoxin encoding gene (*hicB*) is located downstream of the cognate toxin encoding gene (*hicA*) [16]. Several functions have been proposed for HicAB systems including biofilm formation, maintaining MGEs, bacterial persistence, and phage defence, but their biological roles remain largely elusive [18,25].

In this study, a *hicAB* locus was investigated in *N. gonorrhoeae*, and its prevalence across gonococcal genomes was determined. *E. coli* kill/rescue assays showed that gonococcal HicA exerts a bacteriostatic effect that could be counteracted by HicB expression. HicA overexpression in *N. gonorrhoeae* caused growth arrest and a reduction in the number of culturable cells. The HicAB system is transcribed in vitro in the gonococcus under the growth conditions tested. Whether the HicAB system has a role in gonococcal pathogenesis should be further investigated, but a role in biofilm formation, as has been shown in other species, could not be demonstrated.

## 2. Materials and Methods

### 2.1. Bioinformatics

The Toxin–Antitoxin Database (TADB v2.0; [26]) was used to identify the *hicAB* system within the *N. gonorrhoeae* strain FA1090 chromosome (NC_002946). To determine prevalence, a list of *N. gonorrhoeae* isolates with associated complete or draft genome sequences was extracted from the PubMLST database (https://pubmlst.org/organisms/neisseria-spp, accessed on 18 June 2020) using default options, except that in the ‘Attribute values list’ the field ‘species (Ribosomal MLST)’ was selected and the value ‘Neisseria gonorrhoeae’ used as a search term. In addition, the search results were further filtered such that the ‘Ribosomal MLST profile’ was ‘complete’ The BLAST feature embedded in PubMLST was used with default settings to extract the closest sequence in each isolate sequence bin to the sequence of interest (GenBank accession no. AAW90256.1 and AAW90255.1, for FA1090 HicA and HicB, respectively). The outputs generated (data on percentage identity, mismatches, and gaps compared to the query, and a FASTA with flanking file containing the sequence of DNA yielding the closest match to the query in each isolate plus 100 bp of flanking sequence) were subsequently interrogated. ExPASy Translate (https://web.expasy.org/translate/, accessed on 14 September 2020) was used to obtain the amino acid sequence of proteins using the DNA sequence provided by the FASTA with flanking file. Truncated proteins (identified by their shorter alignment length, but high amino acid identity) were resolved by close examination of the DNA sequence and translated amino acid sequence for substitutions creating internal stop codons or insertions/deletions leading to frameshifts. Genes of interest were determined to span multiple contigs if the coding sequence was incomplete and no flanking sequence was present in the FASTA output. Clustal Omega v1.2.4 (https://www.ebi.ac.uk/jdispatcher/msa/clustalo, accessed on 2 July 2025) was used to compare multiple protein sequences and highlight amino acid differences within proteins of interest.

### 2.2. Bacterial Strains, Media, and Growth Conditions

*Escherichia coli* JM109 (Promega, Madison, WI, USA) was used as the host strain for plasmid construction. *E. coli* DH5α (Invitrogen, Waltham, MA, USA) was used for expression and site-directed mutagenesis of *hicA*. *E. coli* strains were grown in Lysogeny broth (LB) or on LB agar and incubated at 37 °C for 24 h. Antibiotics were added when appropriate at the following concentrations: ampicillin (100 μg mL^−1^), tetracycline (25 μg mL^−1^), kanamycin (80 μg mL^−1^), and erythromycin (50 μg mL^−1^). *N. gonorrhoeae* strains (Table S1) were cultured on Thayer-Martin agar composed of GC agar base with 1% soluble hemoglobin and 1% Vitox (Thermo Fisher Scientific, Waltham, MA, USA) at 37 °C in air plus 5% (*v*/*v*) CO_2_ for 48 h. When grown in suspension, gonococcal strains were grown in Brain Heart Infusion (BHI) broth with 1% Vitox at 37 °C with shaking at 200 rpm. When appropriate, antibiotics were added to gonococcal cultures at the following concentrations: kanamycin (80 μg mL^−1^) and erythromycin (2 μg mL^−1^).

### 2.3. Inducible Expression of Gonococcal HicA, HicB, and HicAB in E. coli

Fragments corresponding to *hicA, hicB*, and both coding sequences (as one product) were amplified from *N. gonorrhoeae* FA1090 using sequence-specific primers (Table S2). After digestion with EcoRI and PstI, the PCR products were ligated into identically treated pBAD24 [27] to yield pMS1, pMS2, and pMS3, respectively (Table S3). Using pMS1 as template, *hicA* codons encoding Gly22, His24, His29, His40, and Gly58 residues were individually replaced by codons encoding cysteine (for Gly) or alanine (for His) residues, respectively, using the Q5 Site-Directed Mutagenesis Kit (New England BioLabs, Ipswich, MA, USA) following the manufacturer’s instructions (Table S3). For the delayed induction of HicB experiments, *hicB* was amplified from FA1090, and the product was digested with EcoRI and XhoI and ligated into pME6032 [28] to yield pJTM7 (Table S3). The oligonucleotides used are given in Table S2.

To determine toxicity, overnight cultures of *E. coli* DH5α strains were 1:20-diluted in fresh LB containing 1% (*w*/*v*) D(+)-glucose and appropriate antibiotics and incubated until OD_600_ > 0.3. The cultures were then centrifuged, resuspended in pre-warmed selective media (no glucose), and equilibrated to OD_600_~0.3, after which 1% (*w*/*v*) L(+)-arabinose or D(+)-glucose was added, where required, to induce or repress gene expression. Cultures were incubated for 4 h with OD_600_ measurements taken every hour. For delayed induction of HicB experiments, toxicity assays were carried out in the same manner, except that bacteria were removed hourly, serially diluted, and plated onto selective LB agar ±1 mM IPTG. Following overnight incubation at 37 °C, colonies were counted to determine cfu mL^−1^. All growth curve experiments were repeated on at least three independent occasions.

### 2.4. Generation of a Gonococcal hicAB Mutant

A 2959 bp synthetic linear DNA fragment (GeneArt Strings™; Invitrogen) comprising a kanamycin resistance cassette (based on the *aphA-3* gene of pJMK30 [29]), flanked by 750 bp of FA1090 *hicA* upstream sequence (and containing a naturally occurring 12 bp DNA uptake sequence [30]), and 767 bp FA1090 *hicB* downstream sequence was used to mutate gonococcal strain FA1090 to generate FA1090Δ*hicAB*. The *hicAB* mutagenesis fragment was introduced into FA1090 by natural spot transformation [31]. The deletion of *hicAB* and replacement with the kanamycin resistance cassette via double crossover recombination on either side on the resistance cassette/*hicAB* locus was confirmed by PCR and DNA sequencing.

### 2.5. Complementation of hicA, hicB, and hicAB

Fragments corresponding to the *hicA, hicB*, and *hicAB* coding sequences were amplified from strain FA1090 using gene-specific oligonucleotides (Table S2) incorporating PacI and SacII sites into the amplified fragments. The PacI/SacII-digested fragments were then introduced into PacI/SacII-digested pMR33 [32] to yield pSS1, pSS2, and pSS3, respectively (Table S3). NheI-linearized plasmids were then used to transform FA1090Δ*hicAB* by spot natural transformation to yield FA1090Δ*hicAB::hicA*, FA1090Δ*hicAB::hicB*, and FA1090Δ*hicAB::hicAB*, respectively (Table S1). The correct insertion of *hicA, hicB*, or *hicAB* under the control of the *lac* promoter alongside an *ermC* resistance cassette via double crossover recombination into the FA1090Δ*hicAB trpB*-*iga* intergenic region was confirmed by PCR and DNA sequencing.

### 2.6. Gonococcal Growth Experiments

Overnight cultures of gonococcal strains were diluted back in fresh BHI broth containing 1% Vitox and grown until early logarithmic growth. After equilibration to OD_600_~0.2, IPTG (0.5 mM final concentration) was added to induce gene expression when appropriate [32]. All cultures were incubated with shaking, with OD_600_ measurements taken regularly. At specific time points, aliquots were removed, serially diluted, and plated onto Thayer-Martin agar. Following incubation for 48 h, colonies were counted and cfu mL^−1^ calculated. All growth curve experiments were repeated on at least three independent occasions.

### 2.7. Total RNA Purification and RT-PCR

Briefly, 2 volumes of RNAprotect bacteria reagent (Qiagen, Hilden, Germany) were added to 0.5 mL bacterial culture. Following enzymatic lysis and proteinase K digestion, total RNA was purified using the RNeasy Mini Kit (Qiagen) according to the manufacturer’s instructions. Residual DNA contamination was eliminated using the RNase-Free DNase set (Qiagen) and confirmed by PCR analysis using extracted total RNA as template. The quantity/quality of total RNA was determined using a NanoDrop 1000 Spectrophotometer (NanoDrop Technologies, Wilmington, DE, USA) and an Agilent 2100 Bioanalyzer with RNA 6000 Pico kit (Agilent Technologies, Santa Clara, CA, USA). Reverse transcription, utilizing random 9 bp primers, was performed using the Omniscript RT Kit (Qiagen) according to the manufacturer’s instructions. cDNA was subsequently used in PCR experiments to confirm gene expression using primers listed in Table S2. Previously described 23S rRNA primers were used to generate positive control products [33]. Genomic DNA (gDNA) was extracted using the GenElute Bacterial Genomic DNA kit (Sigma-Aldrich, St. Louis, MO, USA).

### 2.8. Biofilm Formation Assay

A number of 100 μL volumes of gonococci (10^6^ cfu in BHI broth) were added into the wells of a 96-well microtiter plate. IPTG (0.5 mM final concentration) was added to induce gene expression when appropriate. Biofilms were grown without shaking for 24 h at 37 °C in air plus 5% CO_2_ with five replicate wells per strain. Non-attached cells were removed by washing with PBS. Attached bacteria were fixed with ethanol and, after drying by air, stained with 0.8% crystal violet for 10 min. After rinsing and drying, 100% ethanol was added to dissolve the crystal violet and the OD_560_ was quantified to represent biofilm formation. All biofilm experiments were repeated on at least three independent occasions.

### 2.9. Statistical Analysis

Statistical analyses were carried out using GraphPad Prism (Version 10.4). Where appropriate, the data were analyzed using a two-tailed Student’s *t*-test. Differences were considered statistically significant at *p* < 0.05.

## 3. Results

### 3.1. Identification, Prevalence, and Conservation of Gonococcal HicAB

**Figure 1:**
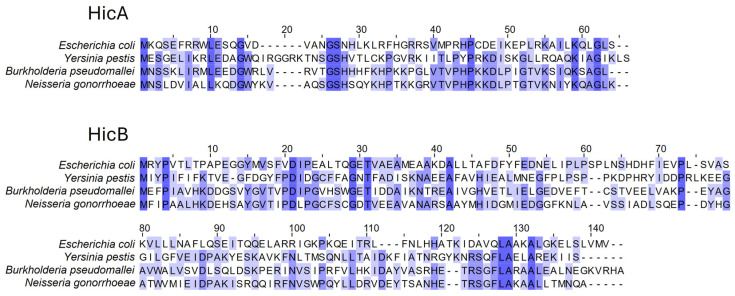
Sequence alignments of the *N. gonorrhoeae* HicA and HicB proteins with the related characterized homologs. The 100% conserved residues are highlighted in dark blue; the 67% conserved residues are highlighted in mid-blue; the 50% conserved residues are highlighted in light blue.

Interrogation of the Toxin–Antitoxin Database (TADB) revealed the presence of a putative *hicAB* TA system corresponding to locus tags *ngo1628*/*ngo*_*RS08085* (*hicA*) and *ngo1627/ngo_RS08080* (*hicB*), respectively, within a region of the *N. gonorrhoeae* FA1090 chromosome previously predicted to encode an incomplete dsDNA phage genome (NgoΦ3; [34]). Additional bioinformatic analysis confirmed the similarity of the putative HicA and HicB proteins with characterized homologs in other bacterial species (Figure 1). For example, the 60 aa gonococcal HicA and 133 aa HicB proteins exhibit 63% and 42% amino acid identity, respectively, to the corresponding proteins of *B. pseudomallei* and 33% and 29% identity, respectively, to the corresponding proteins of *Y. pestis*. To determine the prevalence of the genes in *N. gonorrhoeae*, tBlastN searches using the FA1090 sequences were performed against draft and complete *N. gonorrhoeae* genome sequences available in the PubMLST database [35]. Approximately 84% (4592/5468) of gonococcal strains harboured *hicAB* genes, with 95.5% and 98.8% of *hicAB*-positive strains predicted to encode full-length proteins 100% identical to the FA1090 HicA and HicB proteins, respectively (Tables S4 and S5).

### 3.2. Overexpression of Gonococcal HicA in E. coli Is Bacteriostatic

**Figure 2:**
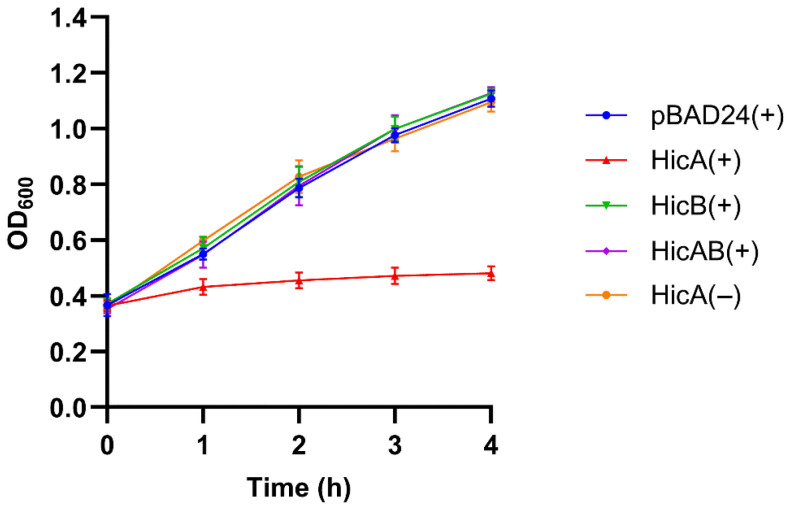
Growth analysis of *E. coli* DH5α expressing gonococcal HicA, HicB, or HicAB. Cultures were equilibrated to OD_600_ of ~0.3 and grown for 4 h following induction with (+) or without (−) arabinose at 0 h. Growth was monitored by OD_600_ measurement and compared to the negative control strain *E. coli* DH5α (pBAD24). The gonococcal HicA toxin inhibited growth, but not in the absence of an inducing agent or when co-expressed with the HicB antitoxin. Data are expressed as the mean ± SD of ≥3 independent experiments.

DNA fragments corresponding to *hicA*, *hicB*, and both genes were amplified from *N. gonorrhoeae* FA1090, ligated into the arabinose-inducible expression plasmid pBAD24 and transformed into *E. coli*. Arabinose-induced *E. coli* DH5α (pMS2), expressing the gonococcal HicB antitoxin, showed no difference in growth compared to the control (Figure 2). However, when the HicA toxin was induced in *E. coli* DH5α (pMS1), bacterial growth was inhibited. Importantly, HicA-mediated growth inhibition was not evident in the absence of arabinose or when expression of both genes was induced.

**Figure 3:**
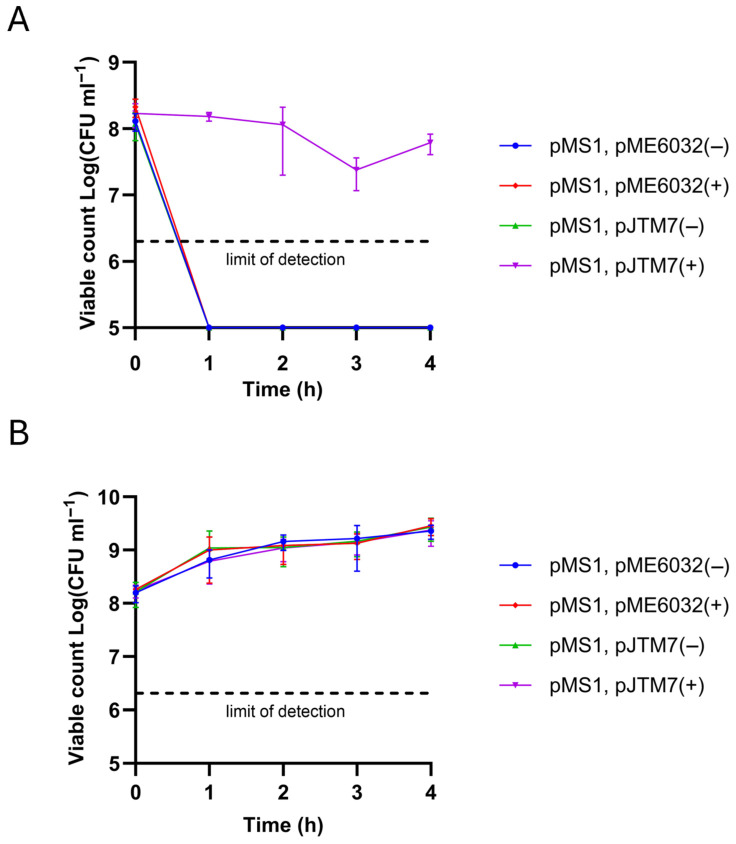
Delayed expression of gonococcal HicB rescues HicA-mediated growth arrest in *E. coli*. Following induction (**A**) or not (**B**) with arabinose for up to 4 h, dilutions of *E. coli* DH5α cultures containing co-resident arabinose-inducible pMS1 (HicA), and IPTG-inducible pJTM7 (HicB) or empty pME6032 plasmids were plated on selective LB plates with (+) or without (−) IPTG and incubated at 37 °C overnight for the determination of colony-forming units. Data are expressed as the mean ± SD of ≥3 independent experiments. For clarity, values below the limit of detection in panel A are plotted at 1 × 10^5^ cfu mL^−1^.

To determine whether overproduction of HicA conferred death or stasis, a delayed induction of a HicB strategy (expressed from a pME6032-based construct, named pJTM7, following induction on IPTG plates) was utilized to rescue cells following exposure to HicA (expressed from pMS1 in broth as before). This demonstrated the ability of HicB to rescue cells from extended exposure to HicA, whereas in the absence of antitoxin, levels of recoverable HicA-exposed cells rapidly fell below the limit of detection (Figure 3A). In contrast, without arabinose induction of HicA expression, all strains grew as normal with or without induction of HicB expression (Figure 3B). The data confirm that the growth arrest response resulting from overproduction of gonococcal HicA is bacteriostatic and can be alleviated by expression of gonococcal HicB.

### 3.3. HicA Amino Acid Residues Required for Toxicity

**Figure 4:**
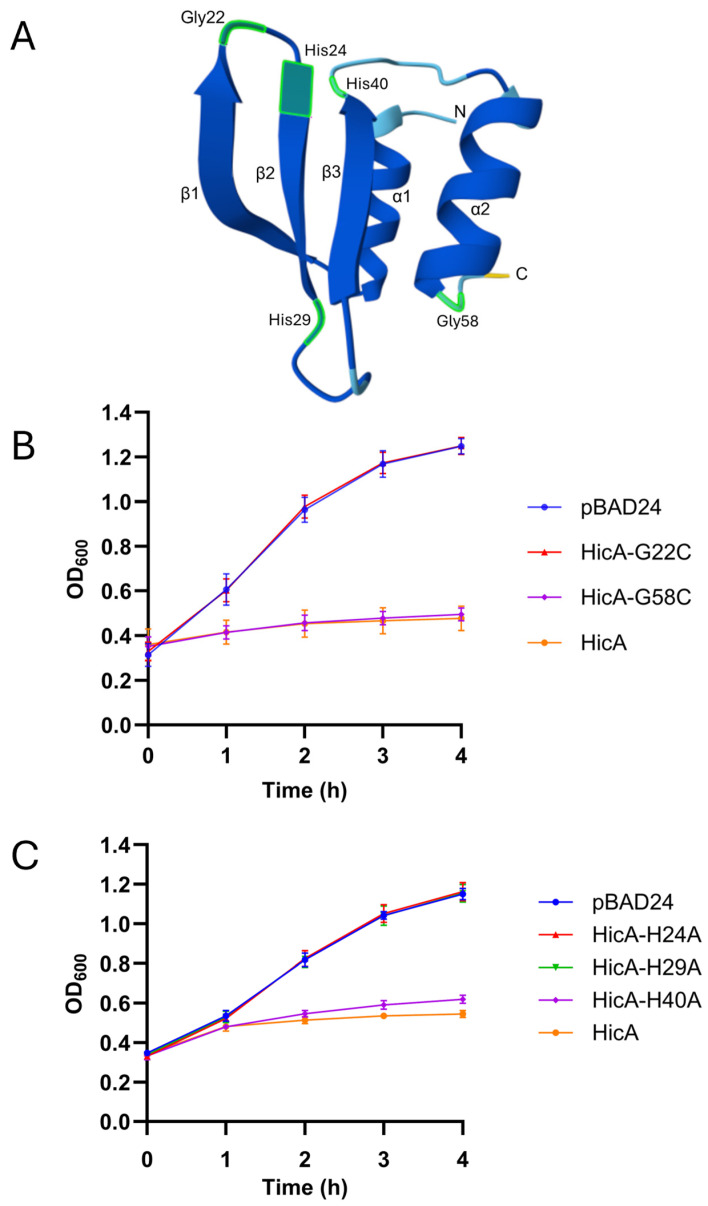
Site-directed mutagenesis of key histidine and glycine residues (Gly22, His24, or His29) abolishes HicA-mediated *E. coli* growth arrest. (**A**) Predicted structure of the *N. gonorrhoeae* FA1090 HicA protein (AF-Q5F6D1-F1) with the five residues (Gly22, His24, His29, His40, and Gly58) selected for mutagenesis highlighted. The *N*- and *C*-termini and secondary structure elements are also indicated. (**B**) Growth analysis of *E. coli* DH5α expressing HicA-G22C or HicA-G58C. (**C**) Growth analysis of *E. coli* DH5α expressing HicA-H24A, HicA-H29A, or HicA-H40A. For growth analysis, cultures were equilibrated to OD_600_ ~0.3 and expression induced at 0 h by the addition of L-arabinose. Growth, as judged by OD_600_ measurement, was compared to the negative control strain *E. coli* DH5α (pBAD24). Data are expressed as the mean ± SD of ≥3 independent experiments.

The AlphaFold Structural database contains predicted FA1090 HicA and HicB structures (AF-Q5F6D1-F1 and AF-Q5F6D2-F1, respectively) with very high average pLDDT scores (91.23 and 93.15, respectively). These suggest that gonococcal HicB contains a partially degraded RNase H fold (comprising an α-helix and three β-sheets) at its *N*-terminal domain and a *C*-terminal ribbon-helix-helix (RHH) DNA-binding domain, whilst gonococcal HicA adopts the distinctive α1β1β2β3α2 fold characteristic of the double-stranded RNA-binding domain (dsRBD) that is conserved in the HicA family (Figure 4A). In other HicA family proteins, highly conserved histidine and glycine residues located in the vicinity of the turn between the first and second *β*-strands have been shown to play a role in toxicity (e.g., [21,23,36]). To examine this in gonococcal HicA, the relevant residues, Gly22 and His24, were changed to cysteine and alanine residues, respectively. Identical amino acids, but in other positions (His29, His40, and Gly58), were also substituted as controls. Growth analysis confirmed that, as expected, the Gly22 and His24 residues were essential for toxicity (Figure 4B,C). HicA-H40A and HicA-G58C conferred similar toxicity as wild-type HicA, but surprisingly, substitution of His29, a residue located in the turn between the second and third *β*-strands, also abolished HicA toxicity (Figure 4C).

### 3.4. Characterization of the HicAB System in N. gonorrhoeae FA1090

**Figure 5:**
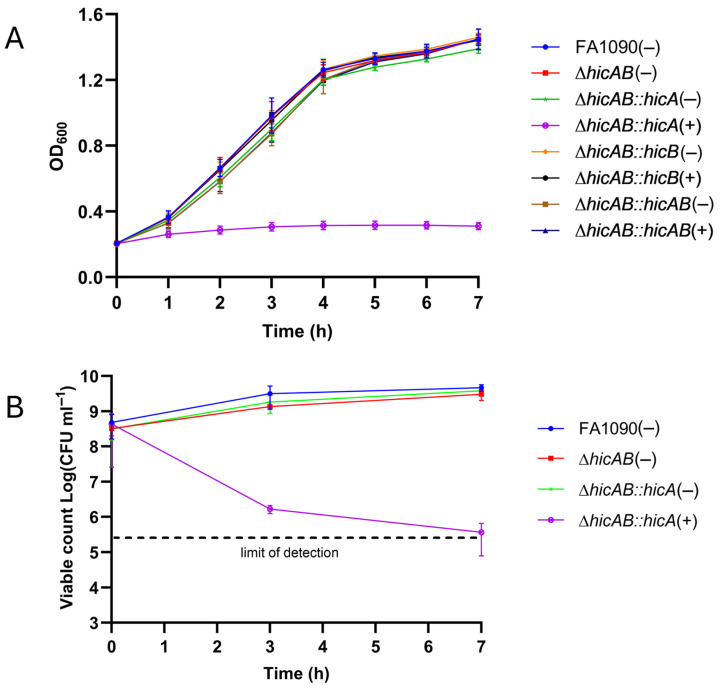
Over-expression of HicA results in gonococcal growth arrest. (**A**) Growth analysis of FA1090∆*hicAB* derivatives expressing HicA, HicB, or both proteins. Cultures were equilibrated to OD_600_ ~0.2. Growth, as judged by OD_600_ measurement, was compared to the wild-type FA1090 control strain following induction with (+) or without (−) IPTG at 0 h. (**B**) Following growth with (+) or without (−) IPTG for 0, 3, or 7 h, dilutions of relevant FA1090 derivatives were plated on Thayer-Martin agar and incubated at 37 °C for 48 h for the determination of colony-forming units. Differences in viable count between FA1090Δ*hicAB::hicA*(+) and the other strains were statistically significant at 7 h (Student’s *t*-test; *p* < 0.05). Data in both panels are expressed as the mean ± SD of ≥3 independent experiments.

Both toxin and antitoxin-encoding genes were deleted and replaced by a kanamycin cassette yielding *N. gonorrhoeae* FA1090Δ*hicAB*. Subsequently, complemented derivatives in which *hicA, hicB* or both genes, under the control of the IPTG-inducible *lac* promoter, and inserted into the FA1090∆*hicAB* chromosome between the *trpB* and *iga* genes, were generated. The in vitro growth characteristics of the complemented strains, with or without IPTG induction, were examined. The complemented strains grew as wild-type, with induction or not, except for IPTG-induced FA1090∆*hicAB*::*hicA* which exhibited growth arrest, consistent with HicA-mediated toxicity, as judged by OD measurements (Figure 5A). Cfu counts of relevant strains showed reduced recovery of viable FA1090∆*hicAB::hicA* following IPTG induction compared to the non-induced and control strains, confirming HicA toxicity (Figure 5B).

The transcription of the *hicAB* genes was examined by extraction of total RNA and reverse transcription polymerase chain reaction (RT-PCR) analysis to detect specific mRNA transcripts. As expected, *hicA*-specific primers yielded an amplification product from IPTG-induced FA1090Δ*hicAB::hicA* (Figure S1). Irrespective of IPTG-induction, *hicA*- and *hicB*-specific transcripts could be detected from cultures of wild-type FA1090 confirming active transcription under in vitro growth conditions. The transcription of *hicA* and *hicB* on a single transcript was confirmed by the amplification of a product spanning both genes (Figure S1).

**Figure 6:**
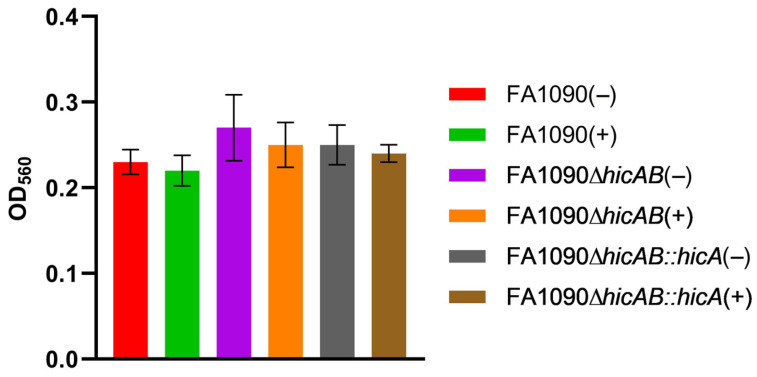
Gonococcal HicAB does not influence biofilm formation. Biofilm formation of FA1090 and derivatives in the presence (+) or absence (−) of IPTG inducer was assessed by crystal violet staining after static growth at 37 °C. No statistically significant differences were observed compared to the wild type grown under matching conditions (Student’s *t*-test; *p* > 0.05). Data are expressed as the mean ± SEM of ≥3 independent experiments.

A role for HicAB systems in biofilm formation has been confirmed in some bacterial species (e.g., *B. pseudomallei* [37]), but excluded in others (e.g., *Pseudomonas aeruginosa* [38]). The ability of wild-type FA1090 and FA1090Δ*hicAB* to form biofilms was compared using a crystal violet biofilm assay. FA1090∆*hicAB::hicA* (IPTG-induced and uninduced) was also included to detect any specific role of the HicA toxin. The results showed no significant differences between all strains (Figure 6) suggesting that the HicAB system is not involved in gonococcal biofilm formation in strain FA1090 under the conditions utilized.

## 4. Discussion

Although TA systems were initially discovered as plasmid-stabilizing elements, they have since been shown to influence several processes relating to bacterial physiology [9,10] and, as such, have become targets for the development of new antimicrobial agents [7,39]. Utilizing a kill/rescue approach, our study demonstrated a bacteriostatic effect following gonococcal HicA expression in *E. coli* which could be alleviated by subsequent HicB expression. This agrees with findings from other HicAB systems following heterologous expression (e.g., [20,22,38]). We also showed that HicA overexpression in *N. gonorrhoeae* causes growth arrest and a reduction in the number of culturable cells, whereas co-expression of the *hicAB* genes under the control of the same IPTG-inducible promoter resulted in no effect. The generation of a FA1090Δ*hicAB::hicA* derivative containing *hicB* under the control of a different inducible promoter, thus allowing delayed expression of HicB following HicA exposure, would enable experimental confirmation that the effect of HicA expression in gonococci is, as in *E. coli*, bacteriostatic.

HicA family proteins are endoribonucleases which have positively charged surfaces and adopt a distinctive α1β1β2β3α2, dsRBD fold-like structure [21]. Interactions between dsRBDs and RNA occur via three regions: α1 with a minor groove of RNA; the loop between β1 and β2 with the next RNA minor groove; and α2 (and some preceding loop residues) with the intervening RNA major groove (e.g., PDB 1DI2 [40]). In line with this, site-directed mutagenesis studies have shown that a catalytic histidine residue positioned at the start of the β2 strand in HicA family proteins is essential for RNase activity (e.g., [17,21,22]). Our data confirm that the corresponding residue (His24) in gonococcal HicA is essential for activity. Likewise, Gly22, essential for HicA toxicity in *B. pseudomallei* HicA [21], is also required for gonococcal HicA toxicity. The mutation of the same amino acids in other parts of the gonococcal HicA protein, His40 (located after β3, but close in space to His24), and Gly58 (located after α2) had no effect on toxicity. Surprisingly, the mutation of gonococcal HicA His29 (located after β2) abolished *E. coli* growth arrest. A histidine residue in this position is not universally conserved across HicA family proteins; however, the NMR structure of *B. pseudomallei* HicA (PDB 4C26), which does harbour His29, reveals a hydrogen bond formed between the His29 imidazole ring and Asp13 located at the end of α1 [21]. This hydrogen bonding pattern is also shown in the predicted structure of FA1090 HicA and supports a hypothesis that His29 substitution sufficiently alters HicA conformation to disrupt target interactions. Purification and subsequent structural characterization studies would confirm this hypothesis and enable the substrate specificities and precise targets of gonococcal HicA to be further understood.

Several *hicAB* loci, including those of *P. aeruginosa* [38], *B. pseudomallei* [20], and *S. pneumoniae* [23], are found in prophage islands. Likewise, the *N. gonorrhoeae hicAB* genes localize to a region of the FA1090 chromosome previously predicted to encode an incomplete prophage genome (NgoΦ3) [34]. Recently, an in silico re-examination concluded that the NgoΦ3 island might, in fact, produce phages [41]. In support of this, the conditional repression of *ngo1630*, encoding for the putative NgoΦ3 cI transcriptional repressor, led to increased expression of several NgoΦ3 genes, the release of phage particles, and cell death [41]. The environmental stimuli triggering phage induction in vivo (if any) remain unclear [41]. Significantly, the *hicAB* genes were not differentially expressed following *ngo1630* knockdown [41], suggestive of functions unrelated to phage production and release. However, the association of some *hicAB* loci with prophage islands may suggest a role in the maintenance of the prophage genes in the chromosome during dormancy. Alternatively, based on the genetic contexts of HicA toxin domains, a role in anti-phage defence for HicAB systems has been proposed [25].

Although the biological roles of many HicAB systems remain elusive, some have been reported to play a role in biofilm formation [18]. For example, in extraintestinal pathogenic *E. coli* (ExPEC), biofilm formation was significantly reduced in a *hicAB* mutant, in a phenotype linked to changes in the expression of outer membrane proteins and cellulose synthesis [19]. Likewise, in *B. pseudomallei*, the deletion of *hicA* resulted in a significant reduction in biofilm formation [37]. In contrast, the deletion of *hicAB* in *P. aeruginosa* had no effect on the biofilm formation [38]. *N. gonorrhoeae* can form biofilms on abiotic surfaces, epithelial cells in vitro, and in the female cervix [42,43]. Our findings, using a crystal violet microtiter plate biofilm assay, suggest that the gonococcal HicAB system is also not involved in biofilm formation. However, different strain backgrounds and experimental approaches (e.g., utilizing continuous-flow chambers or cervical cell monolayers) may reveal a role for HicAB in gonococcal biofilm formation under these specific conditions.

Our study extends understanding of the type II TA systems of *N. gonorrhoeae.* Other characterized systems include a chromosomally encoded, VapBC superfamily system, named FitAB (fast intracellular trafficking) [44,45,46], and a plasmid-based epsilon/zeta TA system (ngε_1/ngζ_1) [47]. The gonococcal ngζ_1 toxin supports plasmid maintenance by converting the UDP-activated sugar precursors required for cell wall synthesis into dead-end products leading to cell death [48]. Recently, a *P. aeruginosa*-derived secondary metabolite, 2-nonyl-4-quinolone *N*-oxide (NQNO), was shown to significantly reduce gonococcal numbers during experimental infection of mice [49]. NQNO-mediated inhibition of the electron transport chain resulted in increased reactive oxygen levels, triggering the degradation of the ngε_1 antitoxin and the release of the ngζ_1 toxin, thus illustrating the potential of subverting TA systems for treating bacterial infections such as gonorrhea [49].

In summary, we provide evidence that *N. gonorrhoeae* expresses a functional HicAB TA system capable of influencing bacterial growth and viability. Understanding the cellular targets, activation, and biological roles of *N. gonorrhoeae* TA systems, including HicAB, might be beneficial in terms of developing novel and effective strategies to control this pathogen.

## Data Availability

The original contributions presented in the study are included in the article and supplementary material. Further inquiries can be directed to the corresponding author.

## Supplementary Materials

The following supporting information can be downloaded at: https://www.mdpi.com/article/10.3390/microorganisms13071619/s1, Table S1: Gonococcal strains used in this study; Table S2: Oligonucleotides used in this study; Table S3: Plasmids used in this study; Table S4: Comparison of HicA encoded by FA1090 (isolate id 2855) to HicA sequences associated with 5468 gonococcal isolate records extracted from PubMLST; Table S5: Comparison of HicB encoded by FA1090 (isolate id 2855) to HicB sequences associated with 5468 gonococcal isolate records extracted from PubMLST. Figure S1: RT-PCR analysis confirms transcription of gonococcal *hicAB* in vitro. References [27,28,32,33] are cited in the Supplementary Materials.
