# Supplementary material for: The Characterization of a Gonococcal HicAB Toxin–Antitoxin System Capable of Causing Bacteriostatic Growth Arrest
Source: Microorganisms. 2025 Jul 9;13(7):1619. doi: 10.3390/microorganisms13071619 (PMC12297915; doi:10.3390/microorganisms13071619)
Supplement: Supplementary file 1 [file microorganisms-13-01619-s001.zip › microorganisms-3713424-supplementary.pdf]

Table S1: Gonococcal strains used in this study

Strain	Characteristics	Source
FA1090	Wild-type strain	ATCC 700825
FA1090Δ*hicAB*	*ngo1628*-*ngo1627* replaced by Kan[r] cassette	This study
FA1090Δ*hicAB::hicA*	FA1090Δ*hicAB* with Erm[r] cassette and *hicA* (*lacPO*) at *iga-trpB*	This study
FA1090Δ*hicAB::hicB*	FA1090Δ*hicAB* with Erm[r] cassette and *hicB* (*lacPO*) at *iga-trpB*	This study
FA1090Δ*hicAB::hicAB*	FA1090Δ*hicAB* with Erm[r] cassette and *hicAB* (*lacPO*) at *iga-trpB*	This study

Table S2: Oligonucleotides used in this study

Oligonucleotide	Sequence[a]	Restriction site
NGO1628F1	CCG**GAATTC**TTGAATAGCCTAGACGTTATTGCC	EcoRI
NGO1628R1	AAA**CTGCAG**TGCCGTTACTTCAAACCGGCTTGC	PstI
NGO1627F1	CCG**GAATTC**ATGTTTATCCCTGCCGCCCTGC	EcoRI
NGO1627R1	AAA**CTGCAG**GGCTATGCCTGATTCATGGTAAGC	PstI
HicA_G22CF1_fwd	TGCACAATCCtGcAGCCATTCGC	
HicA_G22CR1_rev	ACTTTATACCAACCGTCTTG	
HicA_G58CF1_fwd	TAAGCAAGCCtGcTTGAAGTAAC	
HicA_G58CR1_rev	TAGATATTTTTACAGTACCTG	
HicA_H24AF	ATCCGGGAGCgcTTCGCAATATAAG	
HicA_H24AR	TGTGCAACTTTATACCAAC	
HicA_H29AF	GCAATATAAGgcCCCAACAAAAAAAGGCC	
HicA_H29AR	GAATGGCTCCCGGATTGT	
HicA_H40AF	AACCGTGCCGgcCCCGAAAAAAG	
HicA_H40AR	ACACGGCCTTTTTTTGTTG	
NGO1627R2	CATG**CTCGAG**GGCTATGCCTGATTCATGGTAAGC	XhoI
NGO1628comF	CGC**TTAATTAA**TTGAATAGCCTAGACGTTATTGCC	PacI
NGO1628comR	CGC**CCGCGG**TGCCGTTACTTCAAACCGGCTTGC	SacII
NGO1627comF	CGC**TTAATTAA**ATGTTTATCCCTGCCGCCCTGC	PacI
NGO1627comR	CGC**CCGCGG**GGCTATGCCTGATTCATGGTAAGC	SacII
23SF	TGCTTCCAAGCCTTCCAC	
23SR	GAATGGCGTAACGATGGC	

[a] All oligonucleotides were designed from the *N. gonorrhoeae* FA1090 genome sequence except 23SF and 23SR which were previously reported by Mitchev *et al.,* 2022 [1]. Sequences in bold identify restriction enzyme sites. Nucleotides in lower case indicate those used to introduce amino acid substitutions into HicA.

Table S3: Plasmids used in this study

Plasmid	Characteristics	Source [reference]
pBAD24	Arabinose-inducible expression vector; Amp^r	S. Heeb, University of Nottingham, UK [2]
pMS1	*N. gonorrhoeae* FA1090 *hicA* cloned into pBAD24	This study
pMS2	*N. gonorrhoeae* FA1090 *hicB* cloned into pBAD24	This study
pMS3	*N. gonorrhoeae* FA1090 *hicAB* cloned into pBAD24	This study
pAH1	pMS1 derivative encoding HicA-G58C	This study
pAH2	pMS1 derivative encoding HicA-G22C	This study
pSH1	pMS1 derivative encoding HicA-H24A	This study
pSH2	pMS1 derivative encoding HicA-H29A	This study
pSH3	pMS1 derivative encoding HicA-H40A	This study
pME6032	IPTG-inducible expression vector; Tet^r	S. Heeb, University of Nottingham, UK [3]
pJTM7	*N. gonorrhoeae* FA1090 *hicB* cloned into pME6032	This study
pMR33	*iga-trpB* complementation construct (*lacPO*); Kan^r, Erm^r	J. P. Dillard University of Wisconsin, USA [4]
pSS1	*N. gonorrhoeae* FA1090 *hicA* cloned into pMR33	This study
pSS2	*N. gonorrhoeae* FA1090 *hicB* cloned into pMR33	This study
pSS3	*N. gonorrhoeae* FA1090 *hicAB* cloned into pMR33	This study

Table S4: Comparison of HicA encoded by FA1090 (isolate id 2855) to HicA sequences associated with 5468 gonococcal isolate records extracted from PubMLST

% amino acid identity	Number of isolates	Percentage of total isolates	Protein alignment length	Difference in protein sequence compared to FA1090 HicA	Representative isolate (id)
100	4387	80.23	60	None	2855
	9	0.16	53	Truncated due to frameshift	27271
	8	0.15	51	Truncated due to frameshift	47806
98.33	130	2.38	60	1 mismatch (aa39 P→L)	27090
	6	0.11	60	1 mismatch (aa22 G→R)	31572
	4	0.073	60	1 mismatch (aa3 S→N)	47445
	4	0.073	60	1 mismatch (aa56 Q→STOP)	48775
	2	0.037	60	1 mismatch (aa49 T→A)	31641
	2	0.037	60	1 mismatch (aa12 Q→STOP)	32036
	1	0.018	60	1 mismatch (aa22 G→V)	49194
	1	0.018	60	1 mismatch (aa27 Y→H)	54318
98.18	1	0.018	55	Truncated due to frameshift	32029
97.78	1	0.018	45	Truncated due to frameshift	27471
97.06	20	0.37	34	Truncated due to frameshift	32048
96.77	2	0.037	62	Insertion of DG residues at aa12	61761
80	11	0.20	45	Truncated due to frameshift	21072
79.55	2	0.037	44	Truncated due to frameshift	48670
77.78	1	0.018	45	Truncated due to frameshift	32066
58.82	875	16.00	17	Not HicA	21065
45.46	1	0.018	22	Not HicA	61635

Table S5: Comparison of HicB encoded by FA1090 (isolate id 2855) to HicB sequences associated with 5468 gonococcal isolate records extracted from PubMLST

% amino acid identity	Number of isolates	Percentage of total isolates	Protein alignment length	Difference in protein sequence compared to FA1090 HicB	Representative isolate (id)
100	4539	83.01	133	None	2855
99.25	27	0.49	133	1 mismatch (aa43 A→T)	27283
	8	0.15	133	1 mismatch (aa91 Q→STOP)	49199
	8	0.15	133	1 mismatch (aa99 W→STOP)	37102
	3	0.055	133	1 mismatch (aa94 R→STOP)	32064
	2	0.037	133	1 mismatch (aa94 R→Q)	27099
	2	0.037	133	1 mismatch (aa126 A→V)	31759
	2	0.037	133	1 mismatch (aa58 N→K)	31803
	1	0.018	133	1 mismatch (aa96 N→D)	37327
45.74	812	14.85	129	Not HicB	21065
44.96	52	0.95	129	Not HicB	51800
43.81	11	0.20	105	Not HicB	27300
42.70	1	0.018	89	Not HicB	31869

Figure S1: RT-PCR analysis confirms transcription of the gonococcal *hicAB* genes *in vitro.* FA1090 and derivatives of interest were equilibrated to OD_{600} ~0.2 and cultured for 7 h with (+) or without (-) IPTG. Extracted total RNA was used for cDNA synthesis, and cDNA used as template for PCR amplification using *hicA*, *hicB* and 23S rRNA-specific primers. (**A**) *hicA* primers yield a *ca.* 180 bp product from non-induced and induced wild-type FA1090 and IPTG-induced FA1090Δ*hicAB::hicA* confirming *hicA* expression. (**B**) *hicB*-specific primers yield a *ca.* 400 bp product from non-induced and induced wild-type FA1090 confirming *hicB* expression. (**C**) Use of a *hicA* forward and *hicB* reverse primer combination yield a *ca.* 700 bp product confirming the presence of mRNA transcripts spanning both genes in wild-type FA1090. (**D**) Positive control primers confirm 23S rRNA expression in all strains. In all panels, FA1090 genomic (g)DNA was used as a positive control template for amplification and a no template control confirmed the absence of contamination.

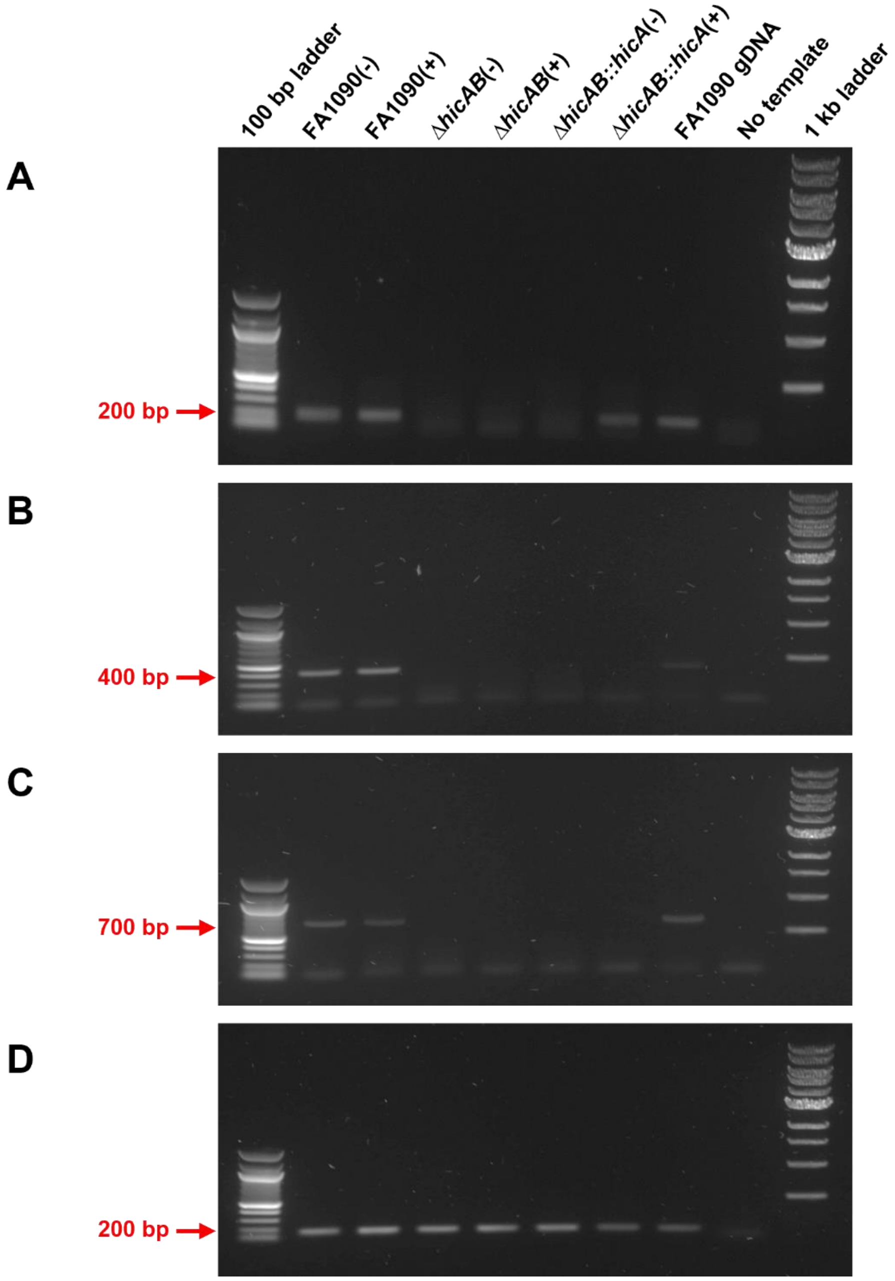

References

1. Mitchev, N.; Singh, R.; Ramsuran, V.; Ismail, A.; Allam, M.; Kwenda, S.; Mnyameni, F.; Garrett, N.; Swe Swe-Han, K.; Niehaus, A.J.; et al. Assessment of antibiotic resistance and efflux pump gene expression in *Neisseria gonorrhoeae* isolates from South Africa by quantitative real-time PCR and regression analysis. *Int J Microbiol* **2022**, *2022*, 7318325, doi:10.1155/2022/7318325.
2. Guzman, L.-M.; Belin, D.; Carson, M.J.; Beckwith, J. Tight regulation, modulation, and high-level expression by vectors containing the arabinose P_{BAD} promoter. *J Bacteriol* **1995**, *177*, 4121-4130, doi:10.1128/jb.177.14.4121-4130.1995.
3. Heeb, S.; Itoh, Y.; Nishijyo, T.; Schnider, U.; Keel, C.; Wade, J.; Walsh, U.; O'Gara, F.; Haas, D. Small, stable shuttle vectors based on the minimal pVS1 replicon for use in gram-negative, plant-associated bacteria. *MPMI* **2000**, *13*, 232-237, doi:10.1094/MPMI.2000.13.2.232.
4. Ramsey, M.E.; Hackett, K.T.; Kotha, C.; Dillard, J.P. New complementation constructs for inducible and constitutive gene expression in *Neisseria gonorrhoeae* and *Neisseria meningitidis*. *Appl Environ Microbiol* **2012**, *78*, 3068-3078, doi:10.1128/AEM.07871-11.
